# Supplementary material for: Impact of green clay authigenesis on element sequestration in marine settings
Source: Nat Commun. 2022 Mar 22;13:1527. doi: 10.1038/s41467-022-29223-6 (PMC8940969; doi:10.1038/s41467-022-29223-6)
Supplement: Supplementary file 1 — Supplementary Information [file 41467_2022_29223_MOESM1_ESM.pdf]

Supplementary Information for "Impact of green clay authigenesis on element sequestration in marine settings"

Andre Baldermann[1]*, Santanu Banerjee[2], György Czuppon[3], Martin Dietzel[1], Juraj Farkaš[4], Stefan Löhr[5], Ulrike Moser[1], Esther Scheiblhofer[1], Nicky M. Wright[6] and Thomas Zack[4,7]

[1]Institute of Applied Geosciences, Graz University of Technology, NAWI Graz Geocenter, Graz, Austria; baldermann@tugraz.at; martin.dietzel@tugraz.at; ulrike.moser@student.tugraz.at; esther.scheiblhofer@student.tugraz.at

[2]Department of Earth Sciences, Indian Institute of Technology Bombay, Powai, Mumbai, India; santanu@iitb.ac.in

[3]Institute for Geological and Geochemical Research, Research Centre for Astronomy and Earth Sciences, Eötvös Loránd Research Network, Budapest, Hungary; czuppon@geochem.hu

[4]Department of Earth Sciences, Metal Isotope Group (MIG), University of Adelaide, North Terrace, Adelaide, Australia; juraj.farkas@adelaide.edu.au

[5]Department of Earth and Environmental Sciences, Macquarie University, Sydney, Australia; stefan.loehr@mq.edu.au

[6]Earthbyte Group, School of Geosciences, University of Sydney, Sydney, Australia; nicky.wright@sydney.edu.au

[7]Department of Earth Sciences, University of Gothenburg, Göteborg, Sweden; thomas.zack@gu.se

Supplementary Figure 1, 2, 3 and 4

Supplementary Table 1, 2, 3, 4 and 5

Study site

The study site is located in a railway outcrop near the village Langenstein within the WNW-ESE-trending Subhercynic Cretaceous Basin, north of the Harz Mountains, set in the Northern German basin[1]. The biostratigraphy of the sedimentary profile is well established based on few index fossils of the Cenomanian (Late Cretaceous), such as the ammonites Mantelliceras dixoni (M. dixoni) and Acanthoceras rhotomagense, the inoceramic bivalves Inoceramus atlanticus and Inoceramus pictus, the oyster Pycnodonte, and planktic foraminifera[2].

The ~15 m thick Langenstein profile was logged at a resolution of ~5 cm. Eight hand-sized samples were collected from the bottom part of the profile (lowermost 5 m) for subsequent petrographic, mineralogical, chemical and isotopic analyses. This interval covers the onset and the progression of the globally expressed Cenomanian transgression, and hosts glauconite-rich strata within the well-constrained M. dixoni Zone of the Lower Cenomanian. The duration of the M. dixoni Zone has been estimated to be 1.8 Myr, lasting from ~97.9 Myr to ~96.1 Myr[3]. The stratigraphic age of the studied interval was verified further by glauconite polytype quantification coupled to K-Ar dating of different grain size sub-fractions of glauconite, which yielded a formation age of 95.0 ± 1.8 Myr[1] for glauconite grains collected at 3.9 m in the profile (top of M. dixoni Zone).

Sample material

Our field observations, together with visual and petrographic inspection of samples, identify five lithofacies across the Langenstein profile (Supplementary Figure 1). Lithofacies 1 (sample S1) occurs at the bottom part of the profile and appears as cross-bedded, arkosic sandstones (so-called Neocomian sandstones) of Lower Cretaceous age. The sandstones comprise sub-rounded quartz, feldspar (albite and orthoclase), mica, chlorite, kaolinite, neomorphic calcite spar, Fe-(hydr)oxides and authigenic illite-smectite that replaces the weathered feldspar grains (Supplementary Figure 1a). The sandstones have a yellowish-grayish brown color. The absence

of marine faunal elements suggests a continental-fluviatile origin of this lithofacies[1,4]. The sandstones are unconformably overlain by a conglomerate (lithofacies 2, samples S2-3), ~30 cm thick. It contains quartz, pebbles of reworked Neocomian sandstones, dolomitic limestone, reworked iron crusts and phosphorite granules, and is consolidated by Fe-(hydr)oxides, mainly hematite cement (Supplementary Figure 1b-c). This poorly exposed lithofacies is devoid of glauconite, so that the lithostratigraphic log of the Langenstein profile is hereby updated. Based on rare findings of siliceous sponge remains and phosphatized fossil fragments, Wilmsen et al. [2] have argued that this basal ironstone conglomerate was deposited on the inner shelf and marks the onset of the transgression at this locality, and thus the change from continental to marine sedimentation. Lithofacies 3 (sample S4) is a sandstone rich in glauconite (~70 wt.%) and silicate detritus (~20 wt.% of mica/illite, feldspar, quartz and lithoclasts), ~40 cm thick, which is weakly consolidated by calcite spar, micrite and Fe-(oxy)hydroxide cement (Supplementary Figure 1d). The glauconitic sandstone was deposited in a proximal shelf setting that developed at the onset of the Cenomanian transgression, as indicated by bioturbation features and marine faunal elements, such as molluscs, brachiopods and benthic foraminifera[5]. Lithofacies 4 appears as glauconitic sandy marlstones with abundant bioclastic debris (samples S5-6) grading into glauconitic highly bioturbated mudstones to packstones with calcispheres and foraminifera (sample S7; Supplementary Figure 1e-g). The glauconitized interval (so-called Glauconitic Pläner Limestones) is marked by a continuous decrease in glauconite content up-section. The total glauconite content is dominated by glauconitized faecal pellets and minor infills within foraminifera chambers (cf. insert in Supplementary Figure 1e). Minor proportions of rounded quartz grains, bio-apatites and bioclasts indicate deposition on the shallow shelf to mid-shelf regions during the Lower to Middle Cenomanian[1,5]. The Glauconitic Pläner Limestones are overlain by marine mudstones and packstones poor in silicate detritus and glauconite (~1 wt.%, lithofacies 5, sample S8; Supplementary Figure 1h; so-called Pläner Limestones), and subsequently deposited glauconite-free mudstones rich in calcareous nannofossils (so-

called Poor rhotomagense Limestone). The limestones were accumulated on mid-shelf to outer shelf settings during the Middle and Upper Cenomanian time[2]. Due to the paucity of glauconite in these limestones, the pelagic facies is not taken into further consideration in this study.

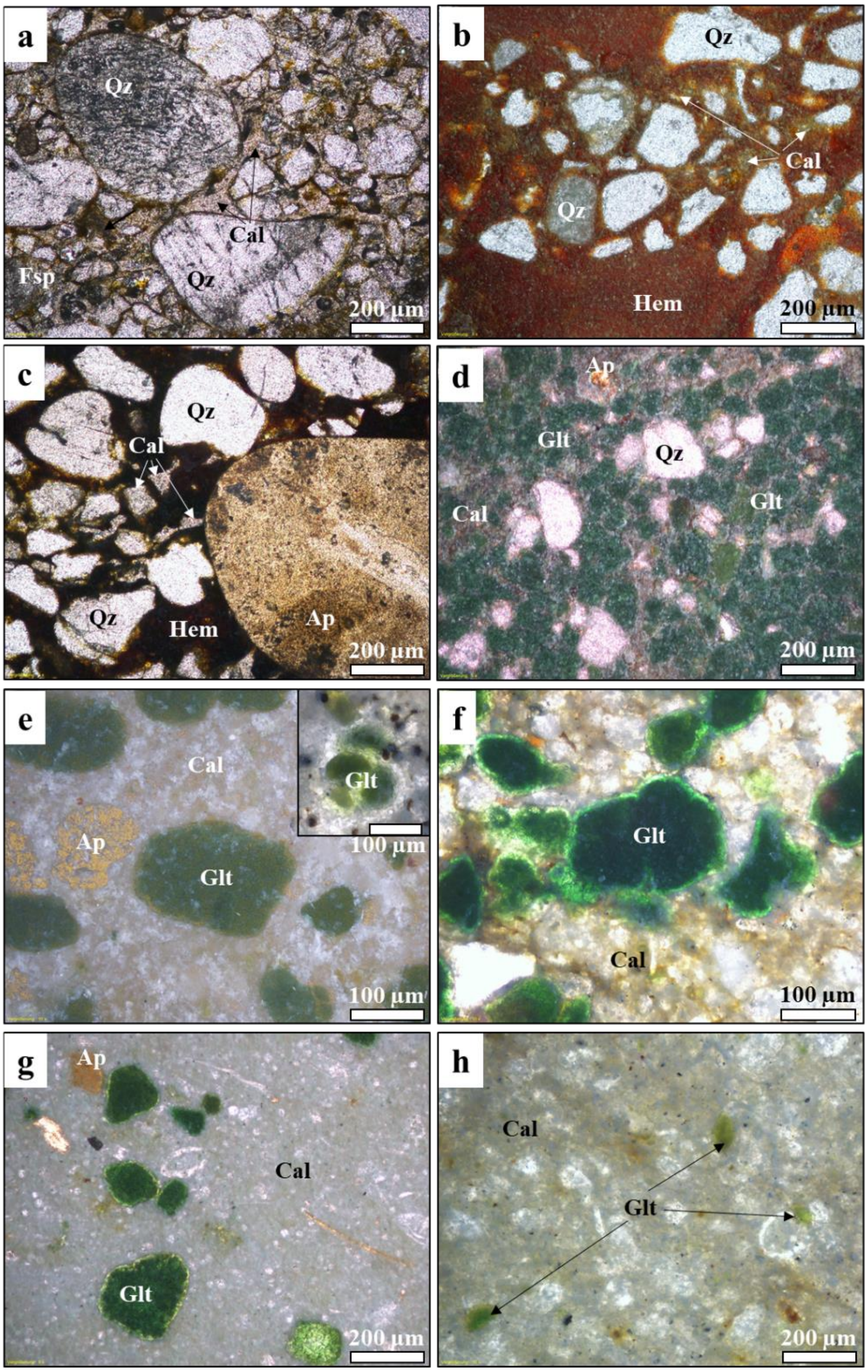

Supplementary Figure 1: Lithofacies at Langenstein. The photomicrographs show a) an arkosic sandstone (‘Neocomian’) made of quartz (Qz), feldspar (Fsp), calcite spar and Fe-(hydr)oxides in a clayey matrix. b-c) Ironstone conglomerate containing quartz, apatite (Ap) and neomorphic calcite hosted in hematite (Hem) cement. d) Glauconite sandstone rich in

glauconitized faecal pellets, which is consolidated by calcitic micrite (Cal) and Fe-(oxy)-hydroxides. e-g) Glauconitic Pläner Limestones appearing as glauconitic bioclastic sandy marl-stones to highly fossiliferous glauconitic bioturbated mudstones to packstones. Note the apatite content and the occurrence of glauconitized foraminifera. h) Pläner Limestones made of marly mud- to packstones. Note the scarcity of glauconite. The overlying Poor rhotomagense Lime-stone are not shown.

Diagenetic history

The aforementioned sedimentary sequence was buried to a maximum depth of 1000-1500 m during the Late Cretaceous, corresponding to a burial temperature of ~50 to 65 °C, as inferred from stable oxygen and carbon isotopic signatures of syntectonic calcite veins and seismic data[1]. Authigenic illite-smectite formed due to burial diagenesis in the terminal Cretaceous (68.0 ± 1.6 Myr, determined by K-Ar dating[1]) and occurs in small quantities within the pore spaces of all rock types. Subsequently, the sedimentary sequence was uplifted in two stages, which occurred during the development of the Harz Thrust Fault in the Lower Coniacian to Campanian and in the Tertiary[6]. Post-depositional alteration of the glauconite-bearing strata, such as (sub)recent surface weathering or local soil formation, is negligible[1].

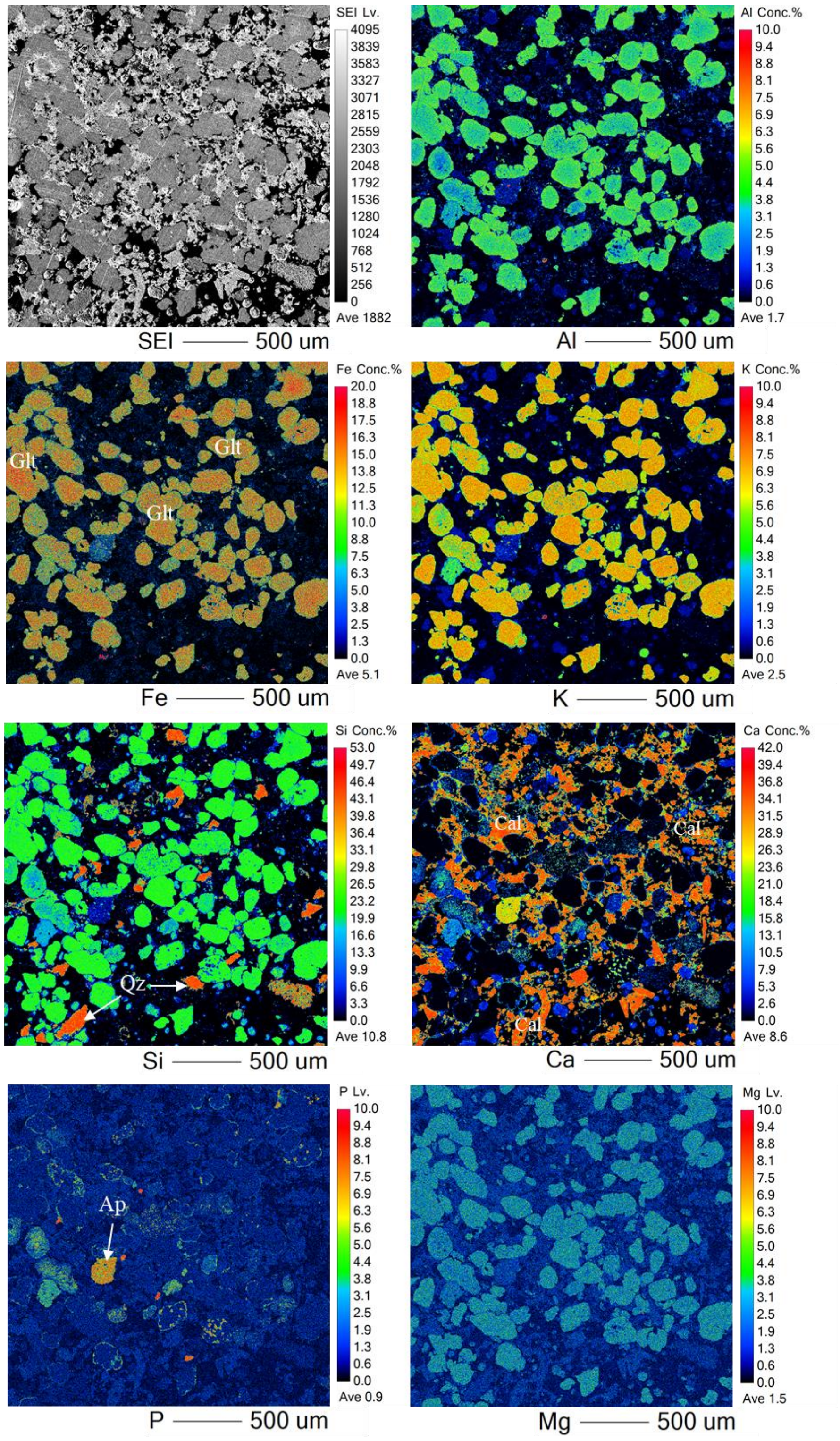

Supplementary Figure 2: The spatial element distribution maps of Al, Fe, K, Si, Ca, P and Mg, as well as the SEI image of sample P5 (lower part of the Glauconitic Pläner Limestones) show glauconite (Glt) in high abundance and a few quartz (Qz) and bio-apatite (Ap) grains hosted in a calcite matrix (Cal). Note that the majority of the glauconites has a homogenous composition.

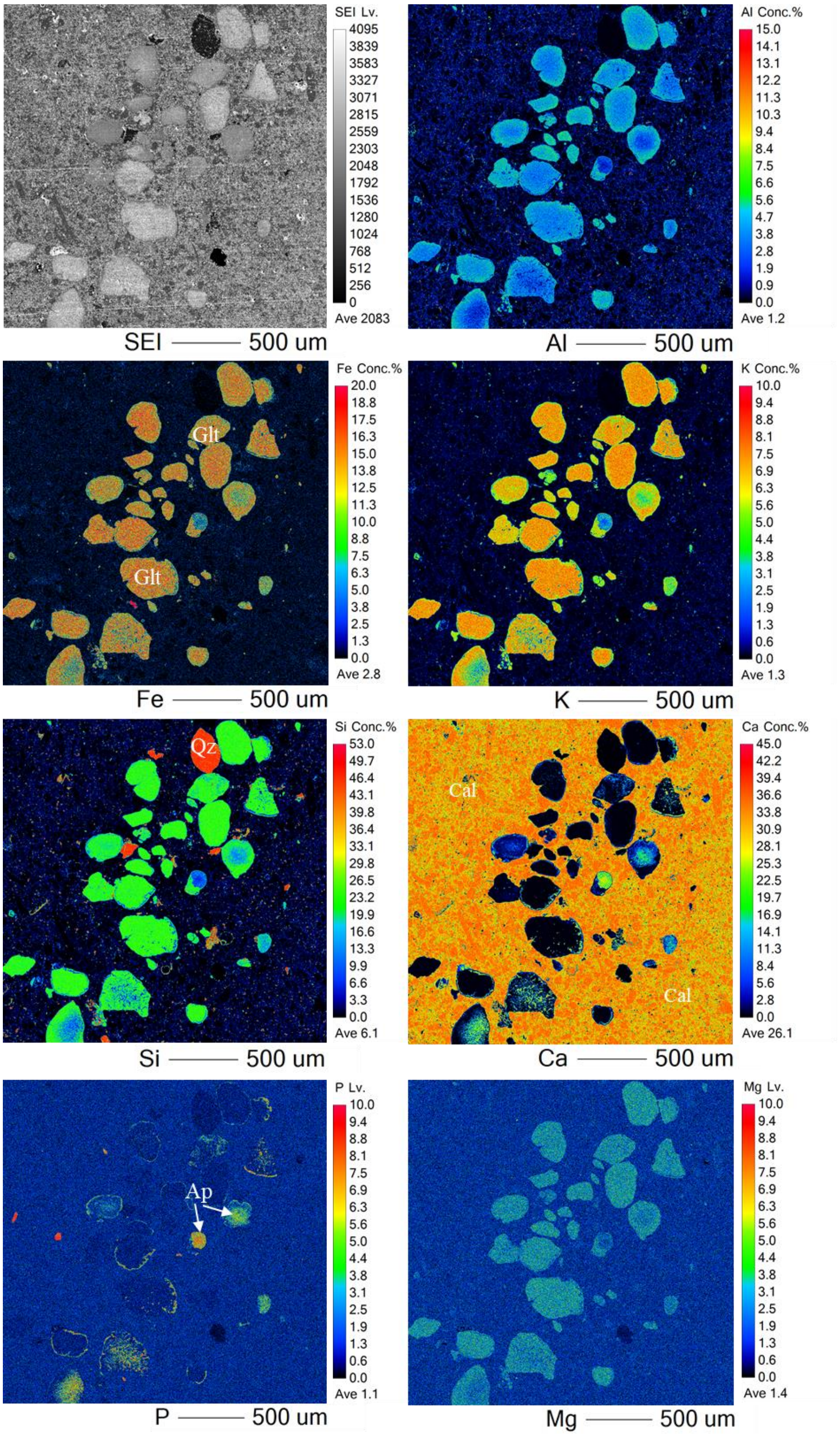

Supplementary Figure 3: The spatial element distribution maps of Al, Fe, K, Si, Ca, P and Mg, as well as the SEI image of sample P6 (middle part of the Glauconitic Pläner Limestones) show glauconite (Glt) in moderate abundance and a few quartz (Qz) and bio-apatite or apatized grains (Ap) hosted in a calcite matrix (Cal). Note that the majority of the glauconites has a homogenous composition.

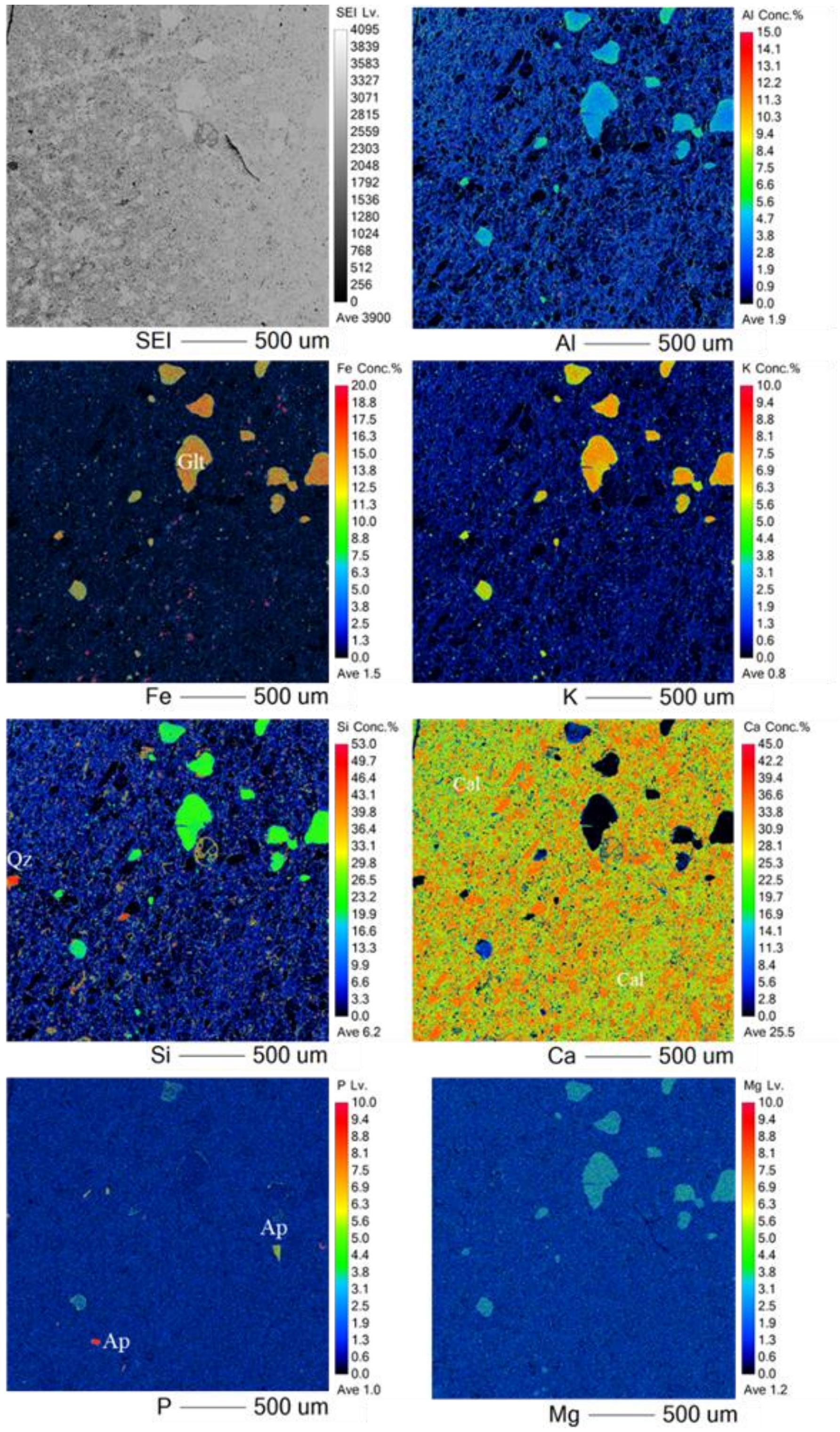

Supplementary Figure 4: The spatial element distribution maps of Al, Fe, K, Si, Ca, P and Mg, as well as the SEI image of sample P7 (uppermost part of the Glauconitic Pläner Limestones) show glauconite (Glt) in low abundance and a few quartz (Qz) and bio-apatite (Ap) grains hosted in a dominant calcite matrix (Cal). Note that the majority of the glauconites has a homogenous composition.

Supplementary Table 1: Chemical composition of the authigenic green grains (glauconite) from the glauconite-sandstone (S4), as well as from the lower (S5), middle (S6) and upper (S7) part of the Glauconitic Pläner Limestones from Langenstein determined by electron microprobe analyses.

Sample code	Al_2O_3 (wt.%)	MgO (wt.%)	SiO_2 (wt.%)	Na_2O (wt.%)	CaO (wt.%)	Fe_2O_3 (wt.%)	FeO (wt.%)	K_2O (wt.%)	P_2O_5 (wt.%)	Total (wt.%)
P4-1	8.360	3.780	51.910	0.011	0.110	20.448	1.841	8.640	0.085	95.185
P4-2	8.340	3.780	50.340	0.025	0.138	21.014	1.892	8.430	0.013	93.972
P4-3	8.520	3.820	50.940	0.035	0.125	20.581	1.853	7.920	0.085	93.879
P4-4	8.580	3.850	51.800	0.049	0.123	20.548	1.850	8.620	0.054	95.474
P4-5	8.340	3.520	49.830	0.052	0.091	20.781	1.871	8.450	0.056	92.991
P4-6	8.660	3.560	51.580	0.022	0.132	19.893	1.791	8.160	0.038	93.836
P4-7	7.810	3.370	51.220	0.058	0.154	21.603	1.945	8.100	0.077	94.337
P4-8	8.580	3.590	51.800	0.039	0.141	20.992	1.890	8.310	0.075	95.417
P4-9	8.820	3.580	51.250	0.000	0.183	19.859	1.788	8.140	0.080	93.700
P4-10	8.570	3.790	51.730	0.033	0.120	21.292	1.917	8.360	0.116	95.928
P4-11	9.770	3.830	52.850	0.046	0.307	18.926	1.704	7.950	0.551	95.934
P4-12	8.870	4.020	51.730	0.025	0.091	20.026	1.803	8.520	0.209	95.294
P4-13	9.260	3.510	52.030	0.027	0.152	19.582	1.763	8.140	0.114	94.578
P4-14	9.200	3.800	52.060	0.000	0.116	19.359	1.743	7.640	0.062	93.980
P4-15	8.585	3.455	46.889	0.088	6.343	15.986	1.439	7.441	4.028	94.253
P4-16	8.586	3.718	52.570	0.030	0.418	21.731	1.956	8.551	0.032	97.592
P4-17	7.798	3.605	51.626	0.063	0.395	22.534	2.029	8.096	0.076	96.220
P4-18	8.451	3.700	51.878	0.038	0.383	21.828	1.965	8.819	0.112	97.174
P4-19	9.581	3.876	52.339	0.021	0.391	19.276	1.735	8.068	0.042	95.329
P4-20	9.374	3.937	52.552	0.042	0.314	17.060	1.536	8.738	0.074	93.627
P4-21	9.244	4.131	52.721	0.029	0.339	17.224	1.551	8.575	0.044	93.857
P4-22	9.541	4.246	52.057	0.055	0.334	17.518	1.577	8.568	0.058	93.954
P4-23	9.234	4.125	52.013	0.000	0.456	18.220	1.640	8.444	0.076	94.210
P4_Average	8.786	3.765	51.553	0.034	0.494	19.838	1.786	8.290	0.268	94.814
P4_deviation	0.533	0.227	1.240	0.021	1.281	1.705	0.153	0.347	0.826	1.187

Sample code	Al_2O_3 (wt.%)	MgO (wt.%)	SiO_2 (wt.%)	Na_2O (wt.%)	CaO (wt.%)	Fe_2O_3 (wt.%)	FeO (wt.%)	K_2O (wt.%)	P_2O_5 (wt.%)	Total (wt.%)
P5-1	8.930	4.180	51.120	0.008	0.209	21.292	1.917	9.140	0.067	96.864
P5-2	9.010	4.200	52.310	0.016	0.242	21.747	1.958	8.990	0.092	98.566
P5-3	8.370	4.230	52.600	0.011	0.205	21.647	1.949	9.440	0.101	98.553
P5-4	7.880	3.900	53.070	0.036	0.433	21.692	1.953	9.360	0.188	98.512
P5-5	8.610	4.050	49.170	0.008	0.239	21.581	1.943	9.290	0.056	94.946
P5-6	8.750	3.900	52.730	0.011	0.227	20.959	1.887	9.110	0.067	97.641
P5-7	8.340	4.190	51.020	0.047	0.180	22.092	1.989	9.190	0.071	97.118
P5-8	8.130	3.940	55.230	0.052	0.113	21.070	1.897	8.610	0.056	99.098
P5-9	8.350	4.250	50.220	0.011	0.141	21.492	1.935	9.050	0.085	95.534
P5-10	7.910	3.850	51.180	0.091	0.194	22.614	2.036	9.250	0.063	97.187
P5-11	7.950	4.140	52.650	0.055	0.204	21.614	1.946	8.950	0.092	97.601
P5-12	8.500	4.290	52.700	0.074	0.191	21.548	1.940	9.080	0.081	98.402
P5-13	8.070	3.880	51.110	0.000	0.148	21.770	1.960	9.200	0.056	96.193
P5-14	8.340	3.950	50.070	0.011	1.480	21.470	1.933	8.910	0.704	96.867
P5-15	8.350	4.030	51.430	0.038	0.294	21.970	1.978	9.150	0.105	97.344

P5-16	7.310	3.620	44.110	0.060	7.860	18.893	1.701	7.990	3.830	95.374
P5-17	8.000	3.870	51.560	0.014	0.148	22.114	1.991	8.090	0.081	95.867
P5-18	8.290	3.850	51.970	0.065	0.160	21.092	1.899	8.990	0.108	96.424
P5-19	8.520	3.920	51.560	0.011	0.207	21.237	1.912	9.030	0.069	96.466
P5-20	8.040	4.120	49.340	0.019	1.870	22.514	2.027	8.660	1.126	97.716
P5-21	7.740	3.720	50.430	0.016	0.153	21.159	1.905	9.230	0.056	94.409
P5-22	8.490	3.890	50.660	0.036	0.209	21.914	1.973	8.870	0.036	96.078
P5-23	8.420	4.330	49.470	0.063	1.940	20.392	1.836	8.680	1.077	96.208
P5-24	7.820	3.990	51.520	0.036	0.196	22.347	2.012	8.940	0.056	96.917
P5-25	8.160	4.150	50.710	0.000	0.146	21.503	1.936	8.890	0.094	95.589
P5-26	8.050	4.240	50.640	0.000	0.155	23.325	2.100	8.850	0.096	97.456
P5-27	7.900	3.780	52.870	0.038	0.212	21.270	1.915	9.190	0.078	97.253
P5-28	8.870	4.190	52.620	0.030	0.208	21.503	1.936	9.060	0.034	98.451
P5-29	7.690	4.110	51.860	0.000	0.192	21.870	1.969	8.320	0.114	96.125
P5-30	7.600	3.980	46.160	0.047	4.340	19.881	1.790	8.260	2.276	94.334
P5-31	8.100	3.910	51.180	0.003	0.278	21.925	1.974	9.120	0.134	96.624
P5-32	8.140	4.010	47.720	0.000	0.703	24.069	2.167	8.330	0.236	95.375
P5-33	8.030	3.900	48.710	0.031	0.269	26.124	2.352	8.690	0.111	98.217
P5-34	8.140	4.240	51.900	0.000	0.175	22.381	2.015	8.880	0.083	97.814
P5-35	8.240	3.920	52.010	0.057	0.184	21.559	1.941	8.670	0.103	96.685
P5-36	6.990	3.630	47.170	0.122	5.130	21.148	1.904	7.690	2.790	96.573
P5-37	8.730	4.120	52.110	0.016	0.085	21.170	1.906	8.840	0.045	97.022
P5-38	8.960	3.910	51.310	0.000	0.076	20.515	1.847	8.970	0.128	95.715
P5-39	9.030	4.160	52.900	0.016	0.074	20.859	1.878	8.990	0.103	98.010
P5-40	8.530	4.110	52.440	0.016	0.068	21.092	1.899	9.030	0.101	97.287
P5-41	9.000	4.210	52.390	0.068	0.087	19.981	1.799	8.750	0.106	96.391
P5-42	9.300	4.190	51.670	0.030	0.121	20.515	1.847	9.110	0.106	96.888
P5-43	9.470	4.430	51.610	0.022	0.049	20.226	1.821	9.420	0.085	97.133
P5-44	9.010	4.080	50.690	0.000	0.071	20.392	1.836	9.030	0.083	95.193
P5-45	9.120	4.410	50.420	0.003	0.091	21.048	1.895	8.950	0.103	96.040
P5-46	9.390	4.140	51.220	0.027	0.075	20.570	1.852	8.840	0.052	96.166
P5-47	8.620	3.870	51.560	0.014	0.065	20.248	1.823	8.930	0.054	95.183
P5-48	7.060	4.230	52.720	0.019	0.074	22.936	2.065	9.140	0.094	98.338
P5-49	7.050	4.500	51.700	0.033	0.077	22.414	2.018	9.000	0.072	96.864
P5-50	6.810	4.350	51.660	0.033	0.041	23.658	2.130	9.050	0.076	97.808
P5-51	6.470	4.240	50.040	0.017	0.059	23.947	2.156	8.820	0.116	95.865
P5-52	6.990	4.230	51.930	0.069	0.053	23.413	2.108	8.730	0.148	97.671
P5-53	6.370	3.900	51.030	0.000	0.071	23.713	2.135	8.960	0.027	96.206
P5-54	6.340	4.040	52.380	0.008	0.047	23.880	2.150	8.730	0.130	97.705
P5-55	6.660	4.170	52.830	0.036	0.025	23.647	2.129	9.090	0.121	98.707
P5-56	6.040	3.930	50.850	0.006	0.078	24.602	2.215	8.980	0.080	96.781
P5-57	6.130	3.740	51.810	0.031	0.075	23.980	2.159	9.000	0.025	96.949
P5-58	6.260	4.080	50.480	0.116	0.086	25.524	2.298	8.750	0.060	97.654
P5-59	6.440	3.980	52.120	0.000	0.056	24.180	2.177	9.270	0.029	98.252
P5-60	6.740	4.250	51.920	0.075	0.066	23.169	2.086	9.230	0.121	97.657
P5-61	6.920	4.450	52.730	0.072	0.048	23.713	2.135	9.290	0.049	99.407
P5-62	6.720	4.250	49.790	0.031	0.091	23.813	2.144	9.010	0.063	95.911
P5-63	6.620	4.150	50.840	0.028	0.044	23.613	2.126	9.040	0.038	96.499
P5-64	5.900	3.600	52.480	0.034	0.088	23.802	2.143	8.990	0.058	97.095
P5-65	6.090	3.840	50.820	0.006	0.068	23.658	2.130	8.940	0.051	95.603
P5-66	5.850	4.000	51.790	0.039	0.293	24.657	2.220	8.910	0.139	97.899
P5-67	5.690	3.990	50.650	0.003	0.057	24.458	2.202	9.090	0.094	96.234
P5-68	6.050	4.090	53.000	0.048	0.044	24.513	2.207	9.180	0.067	99.199
P5-69	6.070	4.280	50.660	0.039	0.065	24.835	2.236	8.960	0.087	97.233
P5-70	6.560	4.400	52.440	0.014	0.032	22.880	2.060	9.120	0.090	97.596

P5-71	6.330	4.320	51.480	0.000	0.055	23.513	2.117	9.080	0.070	96.965
P5-72	6.490	4.290	50.800	0.056	0.044	24.013	2.162	9.290	0.108	97.253
P5-73	6.260	4.130	52.870	0.003	0.040	23.736	2.137	9.270	0.079	98.524
P5-74	6.330	3.970	53.000	0.000	0.039	23.369	2.104	8.970	0.090	97.872
P5-75	6.350	3.860	49.320	0.000	0.053	23.747	2.138	9.210	0.051	94.729
P5-76	6.500	4.440	53.290	0.006	0.041	22.947	2.066	9.290	0.083	98.663
P5-77	7.250	4.370	50.640	0.022	0.052	22.414	2.018	9.150	0.025	95.941
P5-78	7.150	4.000	52.260	0.008	0.044	21.859	1.968	9.280	0.081	96.650
P5-79	7.100	4.020	49.590	0.000	0.080	22.747	2.048	9.210	0.049	94.844
P5-80	7.980	4.330	52.260	0.038	0.052	20.881	1.880	9.210	0.063	96.694
P5-81	8.560	4.130	51.410	0.003	0.071	20.548	1.850	9.020	0.058	95.650
P5_Average	7.621	4.081	51.268	0.027	0.399	22.323	2.010	8.966	0.223	96.918
P5_deviation	1.041	0.204	1.627	0.027	1.149	1.473	0.133	0.309	0.583	1.164

Sample code	Al_2O_3 (wt.%)	MgO (wt.%)	SiO_2 (wt.%)	Na_2O (wt.%)	CaO (wt.%)	Fe_2O_3 (wt.%)	FeO (wt.%)	K_2O (wt.%)	P_2O_5 (wt.%)	Total (wt.%)
P6-1	6.530	4.530	52.600	0.000	0.302	23.813	2.144	9.020	0.025	98.964
P6-2	6.870	4.470	53.490	0.080	0.256	23.636	2.128	8.900	0.038	99.868
P6-3	7.820	4.730	54.970	0.008	0.315	21.170	1.906	9.030	0.036	99.985
P6-4	7.520	4.410	51.500	0.000	0.373	22.358	2.013	8.830	0.054	97.058
P6-5	8.310	4.610	53.250	0.055	0.309	21.359	1.923	9.000	0.047	98.863
P6-6	7.670	4.640	51.850	0.003	0.240	22.269	2.005	8.960	0.000	97.637
P6-7	7.270	4.280	50.290	0.000	0.259	23.169	2.086	8.760	0.020	96.134
P6-8	6.620	4.180	51.180	0.017	0.243	23.014	2.072	8.700	0.105	96.130
P6-9	7.020	4.430	52.460	0.022	0.432	22.769	2.050	8.900	0.040	98.123
P6-10	7.290	4.160	50.880	0.014	0.251	22.214	2.000	8.790	0.022	95.621
P6-11	8.120	4.150	52.410	0.038	0.496	21.559	1.941	8.740	0.067	97.522
P6-12	8.370	4.210	53.440	0.071	0.280	21.903	1.972	8.730	0.052	99.028
P6-13	8.810	4.340	51.690	0.000	0.286	20.870	1.879	8.450	0.072	96.397
P6-14	8.120	4.430	51.730	0.036	1.600	20.848	1.877	8.420	0.737	97.797
P6-15	8.210	4.490	53.340	0.025	0.558	21.514	1.937	8.610	0.200	98.884
P6-16	8.770	4.180	52.550	0.016	0.437	21.092	1.899	8.700	0.020	97.665
P6-17	8.560	4.380	53.380	0.011	0.286	20.559	1.851	8.920	0.070	98.016
P6-18	7.970	4.150	52.580	0.022	0.231	20.959	1.887	8.780	0.029	96.607
P6-19	7.660	4.270	52.510	0.041	2.090	21.592	1.944	8.540	1.008	99.655
P6-20	8.230	3.940	51.400	0.000	1.396	20.703	1.864	8.340	0.270	96.143
P6-21	8.010	4.310	52.690	0.000	0.108	22.181	1.997	8.590	0.027	97.913
P6-22	7.370	4.400	53.540	0.028	0.162	22.147	1.994	8.870	0.036	98.546
P6-23	7.440	4.290	53.670	0.038	0.093	21.692	1.953	8.900	0.029	98.105
P6-24	8.430	4.130	52.940	0.038	0.231	21.492	1.935	8.620	0.054	97.870
P6-25	8.850	4.190	52.970	0.000	0.116	20.848	1.877	8.810	0.036	97.697
P6-26	8.820	4.090	53.110	0.003	0.215	20.459	1.842	8.570	0.174	97.282
P6-27	8.760	4.230	53.750	0.011	0.222	20.270	1.825	8.670	0.232	97.971
P6-28	8.790	4.390	53.450	0.035	0.146	20.204	1.819	8.690	0.034	97.557
P6-29	8.290	4.050	51.140	0.033	1.025	20.215	1.820	8.450	1.525	96.548
P6-30	9.100	4.620	53.860	0.014	0.127	21.548	1.940	8.610	0.063	99.882
P6-31	8.890	4.340	52.140	0.000	0.541	20.304	1.828	8.880	0.049	96.971
P6-32	7.400	3.600	46.980	0.052	2.480	19.526	1.758	7.940	4.250	93.986
P6-33	9.200	4.670	53.290	0.000	0.386	19.182	1.727	8.770	0.360	97.584
P6-34	9.610	4.220	53.190	0.000	0.301	18.471	1.663	8.980	0.252	96.687
P6-35	9.570	4.100	51.930	0.011	0.267	19.526	1.758	9.180	0.314	96.655
P6-36	9.550	4.200	50.140	0.000	1.490	17.405	1.567	8.580	1.803	94.735
P6-37	10.170	4.380	52.370	0.000	0.493	19.082	1.718	8.690	0.436	97.338
P6_Average	8.216	4.302	52.396	0.019	0.515	21.133	1.903	8.728	0.340	97.552
P6_deviation	0.875	0.221	1.393	0.021	0.567	1.397	0.126	0.232	0.776	1.364

Sample code	Al_2O_3 (wt.%)	MgO (wt.%)	SiO_2 (wt.%)	Na_2O (wt.%)	CaO (wt.%)	Fe_2O_3 (wt.%)	FeO (wt.%)	K_2O (wt.%)	P_2O_5 (wt.%)	Total (wt.%)
P7-1	7.840	4.540	52.700	0.000	0.090	21.203	1.909	9.380	0.036	97.698
P7-2	7.240	4.300	53.940	0.017	0.088	22.692	2.043	9.370	0.085	99.774
P7-3	7.370	4.490	52.290	0.006	0.088	22.525	2.028	9.100	0.063	97.959
P7-4	8.510	4.460	53.820	0.022	0.101	19.726	1.776	9.430	0.018	97.862
P7-5	7.680	4.400	53.290	0.000	0.134	21.481	1.934	9.210	0.036	98.165
P7-6	8.220	4.510	53.280	0.000	0.099	21.492	1.935	9.110	0.099	98.744
P7-7	9.140	4.290	53.490	0.081	0.128	20.570	1.852	9.360	0.063	98.974
P7-8	8.310	4.400	53.980	0.005	0.084	21.425	1.929	9.300	0.072	99.506
P7-9	9.500	4.200	51.060	0.011	0.761	18.715	1.685	7.620	1.011	94.562
P7-10	7.590	4.190	52.970	0.077	0.080	22.392	2.016	9.370	0.040	98.725
P7-11	6.780	4.420	51.820	0.000	0.105	23.958	2.157	9.210	0.054	98.503
P7-12	8.000	4.360	52.340	0.033	0.069	22.481	2.024	9.250	0.067	98.624
P7-13	7.130	3.940	52.980	0.033	0.095	23.302	2.098	9.300	0.058	98.937
P7-14	7.310	4.360	52.640	0.044	0.085	22.880	2.060	9.240	0.063	98.682
P7-15	7.280	4.250	51.740	0.072	0.097	22.703	2.044	9.320	0.049	97.554
P7-16	7.440	4.240	50.540	0.011	0.078	21.503	1.936	9.250	0.054	95.051
P7-17	7.330	4.500	53.130	0.066	0.083	23.325	2.100	9.250	0.013	99.797
P7-18	7.220	4.360	52.150	0.011	0.120	23.091	2.079	9.170	0.121	98.322
P7-19	7.630	4.150	53.370	0.006	0.165	22.492	2.025	9.270	0.107	99.214
P7-20	7.760	4.400	51.720	0.022	0.073	23.125	2.082	9.190	0.112	98.483
P7-21	7.300	4.040	52.600	0.028	0.088	23.102	2.080	9.280	0.027	98.545
P7-22	7.730	4.140	51.630	0.039	0.098	22.969	2.068	9.210	0.031	97.915
P7-23	7.970	4.040	53.440	0.066	0.108	22.203	1.999	9.180	0.018	99.023
P7-24	7.560	4.110	52.790	0.027	0.099	21.625	1.947	9.210	0.045	97.413
P7-25	7.470	4.150	53.050	0.060	0.121	22.292	2.007	9.390	0.089	98.629
P7-26	7.940	4.200	52.990	0.017	0.128	22.858	2.058	9.070	0.072	99.332
P7_Average	7.740	4.286	52.683	0.029	0.125	22.159	1.995	9.194	0.096	98.307
P7_deviation	0.611	0.163	0.873	0.026	0.131	1.177	0.106	0.334	0.189	1.212

Supplementary Table 2: Measured δ^{13}C and δ^{18}O isotopic compositions of calcite within the Langenstein profile. GLP – Glauconitic Pläner Limestones.

Sample code	Sample type	δ^{13}C (‰, PDB)	δ^{18}C (‰, PDB)	Position in profile (m)
S1	Neocomian sandstone	-0.78	-5.42	-0.50
S2	Basal conglomerate	-7.51	-7.53	0.10
S4-1	Glauconite-sandstone	-6.03	-7.26	0.50
S4-2	Glauconite-sandstone	0.43	-5.02	0.60
S5-1	GLP	1.07	-3.40	1.10
S5-2	GLP	0.95	-3.65	1.60
S6	GLP	1.33	-3.65	2.40
S7	GLP	1.44	-3.91	2.90
S8	Pläner Limestone	2.13	-3.85	3.40

Supplementary Table 3: Element sequestration rates calculated for the authigenic green grains (glauconite) from the glauconite-bearing sandstone (S4) and the Glauconitic Pläner Limestones (S5-7) from Langenstein. The rates were calculated using measured glauconite abundances and compositions across the profile, assuming a constant sedimentation rate of 0.13 $cm \cdot kyr^{-1}$ for S4 and of 7.0 $cm \cdot kyr^{-1}$ for S5-7, and a sediment density of 2.7 $g \cdot cm^{-3}$ (see 4).

Sample code	Position in profile (m)	Glauconite (wt.%)	K (wt.%)	Mg (wt.%)	K burial rate ($mmol \cdot cm^{-2} \cdot kyr^{-1}$)	Mg burial rate ($mmol \cdot cm^{-2} \cdot kyr^{-1}$)
S4-1	0.6	68	6.9	2.3	0.4	0.2
S5-2	1.6	25	7.4	2.5	8.9	4.9
S6	2.4	5	7.2	2.6	1.7	1.0
S7	2.9	1	7.6	2.6	0.4	0.2

Sample code	Al (wt.%)	Si (wt.%)	Fe (wt.%)	Al burial rate ($mmol \cdot cm^{-2} \cdot kyr^{-1}$)	Si burial rate ($mmol \cdot cm^{-2} \cdot kyr^{-1}$)	Fe burial rate ($mmol \cdot cm^{-2} \cdot kyr^{-1}$)
S4-1	4.6	24.1	15.3	0.4	2.0	0.7
S5-2	4.0	24.0	17.2	7.1	40.3	14.6
S6	4.3	24.5	16.3	1.5	8.2	2.8
S7	4.1	24.6	17	0.3	1.7	0.6

Supplementary Table 4: Element sequestration rates associated with glauconite formation from the Holocene to Triassic. The rates were calculated using a constant sedimentation rate between 0.1 and 100 $cm \cdot kyr^{-1}$, a sediment density of 2.7 $g \cdot cm^{-3}$. Glauconite abundance is taken from published literature[7].

Geological period	Glauconite abundance on shelf (wt.%)	Glauconite composition K (wt.%)	2SD	Sedimentation rate ($cm \cdot kyr^{-1}$)	K burial rate ($mmol \cdot cm^{-2} \cdot kyr^{-1}$) avg	2SD
Holocene	5.6	7	1	0.1	0.027	0.004
Plio-Pleistocene	5.5	7	1	0.1	0.026	0.004
Miocene	4.8	7	1	0.1	0.023	0.003
Oligocene	4.8	7	1	0.1	0.023	0.003
Eocene	10.9	7	1	0.1	0.053	0.008
Paleocene	5.5	7	1	0.1	0.026	0.004
Late Cretaceous	15.6	7	1	0.1	0.075	0.011
Early Cretaceous	9.9	7	1	0.1	0.048	0.007
Jurassic	2.7	7	1	0.1	0.013	0.002
Triassic	2.7	7	1	0.1	0.013	0.002

Geological period	Glauconite abundance on shelf (wt.%)	Glauconite composition K (wt.%)	2SD	Sedimentation rate ($cm \cdot kyr^{-1}$)	K burial rate ($mmol \cdot cm^{-2} \cdot kyr^{-1}$) avg	2SD
Holocene	5.6	7	1	1	0.27	0.04
Plio-Pleistocene	5.5	7	1	1	0.26	0.04
Miocene	4.8	7	1	1	0.23	0.03
Oligocene	4.8	7	1	1	0.23	0.03
Eocene	10.9	7	1	1	0.53	0.08
Paleocene	5.5	7	1	1	0.26	0.04
Late Cretaceous	15.6	7	1	1	0.75	0.11
Early Cretaceous	9.9	7	1	1	0.48	0.07
Jurassic	2.7	7	1	1	0.13	0.02
Triassic	2.7	7	1	1	0.13	0.02

Geological period	Glauconite abundance on shelf (wt.%)	Glauconite composition K (wt.%)	2SD	Sedimentation rate ($cm \cdot kyr^{-1}$)	K burial rate ($mmol \cdot cm^{-2} \cdot kyr^{-1}$) avg	2SD
Holocene	5.6	7	1	10	2.7	0.4
Plio-Pleistocene	5.5	7	1	10	2.6	0.4
Miocene	4.8	7	1	10	2.3	0.3
Oligocene	4.8	7	1	10	2.3	0.3
Eocene	10.9	7	1	10	5.3	0.8
Paleocene	5.5	7	1	10	2.6	0.4
Late Cretaceous	15.6	7	1	10	7.5	1.1
Early Cretaceous	9.9	7	1	10	4.8	0.7
Jurassic	2.7	7	1	10	1.3	0.2
Triassic	2.7	7	1	10	1.3	0.2

Geological period	Glauconite abundance on shelf (wt.%)	Glauconite composition K (wt.%)	2SD	Sedimentation rate ($cm \cdot kyr^{-1}$)	K burial rate ($mmol \cdot cm^{-2} \cdot kyr^{-1}$) avg	2SD
Holocene	5.6	7	1	100	27.1	3.9
Plio-Pleistocene	5.5	7	1	100	26.4	3.8
Miocene	4.8	7	1	100	23.4	3.3
Oligocene	4.8	7	1	100	23.4	3.3
Eocene	10.9	7	1	100	52.7	7.5
Paleocene	5.5	7	1	100	26.4	3.8
Late Cretaceous	15.6	7	1	100	75.3	10.8
Early Cretaceous	9.9	7	1	100	48.1	6.9
Jurassic	2.7	7	1	100	13.2	1.9
Triassic	2.7	7	1	100	13.2	1.9

Geological period	Glauconite abundance on shelf (wt.%)	Glauconite composition Mg (wt.%)	2SD	Sedimentation rate (cm·kyr^{-1})	Mg burial rate (mmol·cm^{-2}·kyr^{-1}) avg	2SD
Holocene	5.6	3	1	0.1	0.019	0.006
Plio-Pleistocene	5.5	3	1	0.1	0.018	0.006
Miocene	4.8	3	1	0.1	0.016	0.005
Oligocene	4.8	3	1	0.1	0.016	0.005
Eocene	10.9	3	1	0.1	0.036	0.012
Paleocene	5.5	3	1	0.1	0.018	0.006
Late Cretaceous	15.6	3	1	0.1	0.052	0.017
Early Cretaceous	9.9	3	1	0.1	0.033	0.011
Jurassic	2.7	3	1	0.1	0.009	0.003
Triassic	2.7	3	1	0.1	0.009	0.003

Geological period	Glauconite abundance on shelf (wt.%)	Glauconite composition Mg (wt.%)	2SD	Sedimentation rate (cm·kyr^{-1})	Mg burial rate (mmol·cm^{-2}·kyr^{-1}) avg	2SD
Holocene	5.6	3	1	1	0.19	0.06
Plio-Pleistocene	5.5	3	1	1	0.18	0.06
Miocene	4.8	3	1	1	0.16	0.05
Oligocene	4.8	3	1	1	0.16	0.05
Eocene	10.9	3	1	1	0.36	0.12
Paleocene	5.5	3	1	1	0.18	0.06
Late Cretaceous	15.6	3	1	1	0.52	0.17
Early Cretaceous	9.9	3	1	1	0.33	0.11
Jurassic	2.7	3	1	1	0.09	0.03
Triassic	2.7	3	1	1	0.09	0.03

Geological period	Glauconite abundance on shelf (wt.%)	Glauconite composition Mg (wt.%)	2SD	Sedimentation rate (cm·kyr^{-1})	Mg burial rate (mmol·cm^{-2}·kyr^{-1}) avg	2SD
Holocene	5.6	3	1	10	1.9	0.6
Plio-Pleistocene	5.5	3	1	10	1.8	0.6
Miocene	4.8	3	1	10	1.6	0.5
Oligocene	4.8	3	1	10	1.6	0.5
Eocene	10.9	3	1	10	3.6	1.2
Paleocene	5.5	3	1	10	1.8	0.6
Late Cretaceous	15.6	3	1	10	5.2	1.7
Early Cretaceous	9.9	3	1	10	3.3	1.1
Jurassic	2.7	3	1	10	0.9	0.3
Triassic	2.7	3	1	10	0.9	0.3

Geological period	Glauconite abundance on shelf (wt.%)	Glauconite composition Mg (wt.%)	2SD	Sedimentation rate ($cm \cdot kyr^{-1}$)	Mg burial rate ($mmol \cdot cm^{-2} \cdot kyr^{-1}$) avg	2SD
Holocene	5.6	3	1	100	18.7	6.2
Plio-Pleistocene	5.5	3	1	100	18.2	6.1
Miocene	4.8	3	1	100	16.1	5.4
Oligocene	4.8	3	1	100	16.1	5.4
Eocene	10.9	3	1	100	36.4	12.1
Paleocene	5.5	3	1	100	18.2	6.1
Late Cretaceous	15.6	3	1	100	51.9	17.3
Early Cretaceous	9.9	3	1	100	33.1	11.0
Jurassic	2.7	3	1	100	9.1	3.0
Triassic	2.7	3	1	100	9.1	3.0

Geological period	Glauconite abundance on shelf (wt.%)	Glauconite composition Al (wt.%)	2SD	Sedimentation rate ($cm \cdot kyr^{-1}$)	Al burial rate ($mmol \cdot cm^{-2} \cdot kyr^{-1}$) avg	2SD
Holocene	5.6	4	1	0.1	0.022	0.006
Plio-Pleistocene	5.5	4	1	0.1	0.022	0.005
Miocene	4.8	4	1	0.1	0.019	0.005
Oligocene	4.8	4	1	0.1	0.019	0.005
Eocene	10.9	4	1	0.1	0.044	0.011
Paleocene	5.5	4	1	0.1	0.022	0.005
Late Cretaceous	15.6	4	1	0.1	0.062	0.016
Early Cretaceous	9.9	4	1	0.1	0.040	0.010
Jurassic	2.7	4	1	0.1	0.011	0.003
Triassic	2.7	4	1	0.1	0.011	0.003

Geological period	Glauconite abundance on shelf (wt.%)	Glauconite composition Al (wt.%)	2SD	Sedimentation rate ($cm \cdot kyr^{-1}$)	Al burial rate ($mmol \cdot cm^{-2} \cdot kyr^{-1}$) avg	2SD
Holocene	5.6	4	1	1	0.22	0.06
Plio-Pleistocene	5.5	4	1	1	0.22	0.05
Miocene	4.8	4	1	1	0.19	0.05
Oligocene	4.8	4	1	1	0.19	0.05
Eocene	10.9	4	1	1	0.44	0.11
Paleocene	5.5	4	1	1	0.22	0.05
Late Cretaceous	15.6	4	1	1	0.62	0.16
Early Cretaceous	9.9	4	1	1	0.40	0.10
Jurassic	2.7	4	1	1	0.11	0.03
Triassic	2.7	4	1	1	0.11	0.03

Geological period	Glauconite abundance on shelf (wt.%)	Glauconite composition Al (wt.%)	2SD	Sedimentation rate (cm·kyr^{-1})	Al burial rate (mmol·cm^{-2}·kyr^{-1}) avg	2SD
Holocene	5.6	4	1	10	2.2	0.6
Plio-Pleistocene	5.5	4	1	10	2.2	0.5
Miocene	4.8	4	1	10	1.9	0.5
Oligocene	4.8	4	1	10	1.9	0.5
Eocene	10.9	4	1	10	4.4	1.1
Paleocene	5.5	4	1	10	2.2	0.5
Late Cretaceous	15.6	4	1	10	6.2	1.6
Early Cretaceous	9.9	4	1	10	4.0	1.0
Jurassic	2.7	4	1	10	1.1	0.3
Triassic	2.7	4	1	10	1.1	0.3

Geological period	Glauconite abundance on shelf (wt.%)	Glauconite composition Al (wt.%)	2SD	Sedimentation rate (cm·kyr^{-1})	Al burial rate (mmol·cm^{-2}·kyr^{-1}) avg	2SD
Holocene	5.6	4	1	100	22.4	5.6
Plio-Pleistocene	5.5	4	1	100	21.8	5.5
Miocene	4.8	4	1	100	19.4	4.8
Oligocene	4.8	4	1	100	19.4	4.8
Eocene	10.9	4	1	100	43.7	10.9
Paleocene	5.5	4	1	100	21.8	5.5
Late Cretaceous	15.6	4	1	100	62.3	15.6
Early Cretaceous	9.9	4	1	100	39.8	10.0
Jurassic	2.7	4	1	100	10.9	2.7
Triassic	2.7	4	1	100	10.9	2.7

Geological period	Glauconite abundance on shelf (wt.%)	Glauconite composition Si (wt.%)	2SD	Sedimentation rate (cm·kyr^{-1})	Si burial rate (mmol·cm^{-2}·kyr^{-1}) avg	2SD
Holocene	5.6	24	2	0.1	0.13	0.01
Plio-Pleistocene	5.5	24	2	0.1	0.13	0.01
Miocene	4.8	24	2	0.1	0.11	0.01
Oligocene	4.8	24	2	0.1	0.11	0.01
Eocene	10.9	24	2	0.1	0.25	0.02
Paleocene	5.5	24	2	0.1	0.13	0.01
Late Cretaceous	15.6	24	2	0.1	0.36	0.03
Early Cretaceous	9.9	24	2	0.1	0.23	0.02
Jurassic	2.7	24	2	0.1	0.06	0.01
Triassic	2.7	24	2	0.1	0.06	0.01

Geological period	Glauconite abundance on shelf (wt.%)	Glauconite composition Si (wt.%)	2SD	Sedimentation rate ($cm \cdot kyr^{-1}$)	Si burial rate ($mmol \cdot cm^{-2} \cdot kyr^{-1}$) avg	2SD
Holocene	5.6	24	2	1	1.29	0.11
Plio-Pleistocene	5.5	24	2	1	1.26	0.10
Miocene	4.8	24	2	1	1.12	0.09
Oligocene	4.8	24	2	1	1.12	0.09
Eocene	10.9	24	2	1	2.52	0.21
Paleocene	5.5	24	2	1	1.26	0.10
Late Cretaceous	15.6	24	2	1	3.59	0.30
Early Cretaceous	9.9	24	2	1	2.29	0.19
Jurassic	2.7	24	2	1	0.63	0.05
Triassic	2.7	24	2	1	0.63	0.05

Geological period	Glauconite abundance on shelf (wt.%)	Glauconite composition Si (wt.%)	2SD	Sedimentation rate ($cm \cdot kyr^{-1}$)	Si burial rate ($mmol \cdot cm^{-2} \cdot kyr^{-1}$) avg	2SD
Holocene	5.6	24	2	10	12.9	1.1
Plio-Pleistocene	5.5	24	2	10	12.6	1.0
Miocene	4.8	24	2	10	11.2	0.9
Oligocene	4.8	24	2	10	11.2	0.9
Eocene	10.9	24	2	10	25.2	2.1
Paleocene	5.5	24	2	10	12.6	1.0
Late Cretaceous	15.6	24	2	10	35.9	3.0
Early Cretaceous	9.9	24	2	10	22.9	1.9
Jurassic	2.7	24	2	10	6.3	0.5
Triassic	2.7	24	2	10	6.3	0.5

Geological period	Glauconite abundance on shelf (wt.%)	Glauconite composition Si (wt.%)	2SD	Sedimentation rate ($cm \cdot kyr^{-1}$)	Si burial rate ($mmol \cdot cm^{-2} \cdot kyr^{-1}$) avg	2SD
Holocene	5.6	24	2	100	129.2	10.8
Plio-Pleistocene	5.5	24	2	100	125.9	10.5
Miocene	4.8	24	2	100	111.7	9.3
Oligocene	4.8	24	2	100	111.7	9.3
Eocene	10.9	24	2	100	251.8	21.0
Paleocene	5.5	24	2	100	125.9	10.5
Late Cretaceous	15.6	24	2	100	359.4	29.9
Early Cretaceous	9.9	24	2	100	229.4	19.1
Jurassic	2.7	24	2	100	62.9	5.2
Triassic	2.7	24	2	100	62.9	5.2

Geological period	Glauconite abundance on shelf (wt.%)	Glauconite composition Fe (wt.%)	2SD	Sedimentation rate (cm·kyr^{-1})	Fe burial rate (mmol·cm^{-2}·kyr^{-1}) avg	2SD
Holocene	5.6	18	2	0.1	0.049	0.005
Plio-Pleistocene	5.5	18	2	0.1	0.047	0.005
Miocene	4.8	18	2	0.1	0.042	0.005
Oligocene	4.8	18	2	0.1	0.042	0.005
Eocene	10.9	18	2	0.1	0.095	0.011
Paleocene	5.5	18	2	0.1	0.047	0.005
Late Cretaceous	15.6	18	2	0.1	0.136	0.015
Early Cretaceous	9.9	18	2	0.1	0.087	0.010
Jurassic	2.7	18	2	0.1	0.024	0.003
Triassic	2.7	18	2	0.1	0.024	0.003

Geological period	Glauconite abundance on shelf (wt.%)	Glauconite composition Fe (wt.%)	2SD	Sedimentation rate (cm·kyr^{-1})	Fe burial rate (mmol·cm^{-2}·kyr^{-1}) avg	2SD
Holocene	5.6	18	2	1	0.49	0.05
Plio-Pleistocene	5.5	18	2	1	0.47	0.05
Miocene	4.8	18	2	1	0.42	0.05
Oligocene	4.8	18	2	1	0.42	0.05
Eocene	10.9	18	2	1	0.95	0.11
Paleocene	5.5	18	2	1	0.47	0.05
Late Cretaceous	15.6	18	2	1	1.36	0.15
Early Cretaceous	9.9	18	2	1	0.87	0.10
Jurassic	2.7	18	2	1	0.24	0.03
Triassic	2.7	18	2	1	0.24	0.03

Geological period	Glauconite abundance on shelf (wt.%)	Glauconite composition Fe (wt.%)	2SD	Sedimentation rate (cm·kyr^{-1})	Fe burial rate (mmol·cm^{-2}·kyr^{-1}) avg	2SD
Holocene	5.6	18	2	10	4.9	0.5
Plio-Pleistocene	5.5	18	2	10	4.7	0.5
Miocene	4.8	18	2	10	4.2	0.5
Oligocene	4.8	18	2	10	4.2	0.5
Eocene	10.9	18	2	10	9.5	1.1
Paleocene	5.5	18	2	10	4.7	0.5
Late Cretaceous	15.6	18	2	10	13.6	1.5
Early Cretaceous	9.9	18	2	10	8.7	1.0
Jurassic	2.7	18	2	10	2.4	0.3
Triassic	2.7	18	2	10	2.4	0.3

Geological period	Glauconite abundance on shelf (wt.%)	Glauconite composition Fe (wt.%)	2SD	Sedimentation rate ($cm \cdot kyr^{-1}$)	Fe burial rate ($mmol \cdot cm^{-2} \cdot kyr^{-1}$) avg	2SD
Holocene	5.6	18	2	100	48.7	5.4
Plio-Pleistocene	5.5	18	2	100	47.5	5.3
Miocene	4.8	18	2	100	42.1	4.7
Oligocene	4.8	18	2	100	42.1	4.7
Eocene	10.9	18	2	100	95.0	10.6
Paleocene	5.5	18	2	100	47.5	5.3
Late Cretaceous	15.6	18	2	100	135.6	15.1
Early Cretaceous	9.9	18	2	100	86.5	9.6
Jurassic	2.7	18	2	100	23.7	2.6
Triassic	2.7	18	2	100	23.7	2.6

Supplementary Table 5: Element palaeo-fluxes associated with glauconite formation from the Holocene to the Triassic. Calculations are based on the average elemental sequestration rates (see Table S4), and estimated 'low' and 'high' shelf areas (0-200 m; [†: 8,9]; [‡: 10-12]) through geological time.

Geological period	Glauconite abundance on shelf (wt.%)	Average K burial rate ($mmol \cdot cm^{-2} \cdot kyr^{-1}$)	World shelf area ($m^2 \cdot 10^{12}$) †	2SD	Shallow ocean K palaeo-flux ($Tmol \cdot yr^{-1}$) †	2SD
Holocene	5.6	2.7	17.9	9.2	0.027	0.014
Plio-Pleistocene	5.5	2.6	15.5	3.5	0.022	0.005
Miocene	4.8	2.3	12.5	1.4	0.014	0.002
Oligocene	4.8	2.3	11.9	1.9	0.013	0.002
Eocene	10.9	5.3	21.4	9.3	0.123	0.054
Paleocene	5.5	2.6	23.6	2.6	0.034	0.004
Late Cretaceous	15.6	7.5	25.5	3.9	0.299	0.045
Early Cretaceous	9.9	4.8	25.1	6.0	0.120	0.029
Jurassic	2.7	1.3	16.2	7.8	0.006	0.003
Triassic	2.7	1.3	11.3	8.0	0.004	0.003

Geological period	Glauconite abundance on shelf (wt.%)	Average K burial rate (mmol·cm^{-2}·kyr^{-1})	World shelf area (m^2 ·10^{12}) ‡	2SD	Shallow ocean K palaeo-flux (Tmol·yr^{-1}) ‡	2SD
Holocene	5.6	2.7				
Plio-Pleistocene	5.5	2.6	62.3	0.1	0.090	0.0002
Miocene	4.8	2.3	66.4	4.2	0.075	0.005
Oligocene	4.8	2.3	73.3	1.4	0.083	0.002
Eocene	10.9	5.3	78.1	1.4	0.449	0.008
Paleocene	5.5	2.6	78.8	0.6	0.113	0.001
Late Cretaceous	15.6	7.5	82.2	3.1	0.964	0.036
Early Cretaceous	9.9	4.8	75.9	7.5	0.363	0.036
Jurassic	2.7	1.3	68.9	4.9	0.025	0.002
Triassic	2.7	1.3	56.8	2.3	0.020	0.001

Geological period	Glauconite abundance on shelf (wt.%)	Average Mg burial rate (mmol·cm^{-2}·kyr^{-1})	World shelf area (m^2 ·10^{12}) †	2SD	Shallow ocean Mg palaeo-flux (Tmol·yr^{-1}) †	2SD
Holocene	5.6	1.9	17.9	9.2	0.019	0.010
Plio-Pleistocene	5.5	1.8	15.5	3.5	0.015	0.003
Miocene	4.8	1.6	12.5	1.4	0.010	0.001
Oligocene	4.8	1.6	11.9	1.9	0.009	0.001
Eocene	10.9	3.6	21.4	9.3	0.085	0.037
Paleocene	5.5	1.8	23.6	2.6	0.023	0.003
Late Cretaceous	15.6	5.2	25.5	3.9	0.206	0.031
Early Cretaceous	9.9	3.3	25.1	6.0	0.083	0.020
Jurassic	2.7	0.9	16.2	7.8	0.004	0.002
Triassic	2.7	0.9	11.3	8.0	0.003	0.002

Geological period	Glauconite abundance on shelf (wt.%)	Average Mg burial rate (mmol·cm^{-2}·kyr^{-1})	World shelf area (m^2 ·10^{12}) ‡	2SD	Shallow ocean Mg palaeo-flux (Tmol·yr^{-1}) ‡	2SD
Holocene	5.6	1.9				
Plio-Pleistocene	5.5	1.8	62.3	0.1	0.062	0.0001
Miocene	4.8	1.6	66.4	4.2	0.052	0.003
Oligocene	4.8	1.6	73.3	1.4	0.057	0.001
Eocene	10.9	3.6	78.1	1.4	0.310	0.006
Paleocene	5.5	1.8	78.8	0.6	0.078	0.001
Late Cretaceous	15.6	5.2	82.2	3.1	0.665	0.025
Early Cretaceous	9.9	3.3	75.9	7.5	0.250	0.025
Jurassic	2.7	0.9	68.9	4.9	0.017	0.001
Triassic	2.7	0.9	56.8	2.3	0.014	0.001

Geological period	Glauconite abundance on shelf (wt.%)	Average Al burial rate (mmol·cm^{-2}·kyr^{-1})	World shelf area (m^2 ·10^{12}) †	2SD	Shallow ocean Al palaeo-flux (Tmol·yr^{-1}) †	2SD
Holocene	5.6	2.2	17.9	9.2	0.023	0.012
Plio-Pleistocene	5.5	2.2	15.5	3.5	0.018	0.004
Miocene	4.8	1.9	12.5	1.4	0.012	0.001
Oligocene	4.8	1.9	11.9	1.9	0.011	0.002
Eocene	10.9	4.4	21.4	9.3	0.102	0.044
Paleocene	5.5	2.2	23.6	2.6	0.028	0.003
Late Cretaceous	15.6	6.2	25.5	3.9	0.248	0.037
Early Cretaceous	9.9	4.0	25.1	6.0	0.099	0.024
Jurassic	2.7	1.1	16.2	7.8	0.005	0.002
Triassic	2.7	1.1	11.3	8.0	0.003	0.002

Geological period	Glauconite abundance on shelf (wt.%)	Average Al burial rate (mmol·cm^{-2}·kyr^{-1})	World shelf area (m^2 ·10^{12}) ‡	2SD	Shallow ocean Al palaeo-flux (Tmol·yr^{-1}) ‡	2SD
Holocene	5.6	2.2				
Plio-Pleistocene	5.5	2.2	62.3	0.1	0.074	0.0001
Miocene	4.8	1.9	66.4	4.2	0.062	0.004
Oligocene	4.8	1.9	73.3	1.4	0.069	0.001
Eocene	10.9	4.4	78.1	1.4	0.372	0.007
Paleocene	5.5	2.2	78.8	0.6	0.094	0.001
Late Cretaceous	15.6	6.2	82.2	3.1	0.798	0.030
Early Cretaceous	9.9	4.0	75.9	7.5	0.300	0.030
Jurassic	2.7	1.1	68.9	4.9	0.021	0.001
Triassic	2.7	1.1	56.8	2.3	0.017	0.001

Geological period	Glauconite abundance on shelf (wt.%)	Average Si burial rate (mmol·cm^{-2}·kyr^{-1})	World shelf area (m^2 ·10^{12}) †	2SD	Shallow ocean Si palaeo-flux (Tmol·yr^{-1}) †	2SD
Holocene	5.6	12.9	17.9	9.2	0.131	0.067
Plio-Pleistocene	5.5	12.6	15.5	3.5	0.106	0.024
Miocene	4.8	11.2	12.5	1.4	0.067	0.008
Oligocene	4.8	11.2	11.9	1.9	0.064	0.010
Eocene	10.9	25.2	21.4	9.3	0.587	0.256
Paleocene	5.5	12.6	23.6	2.6	0.162	0.018
Late Cretaceous	15.6	35.9	25.5	3.9	1.428	0.216
Early Cretaceous	9.9	22.9	25.1	6.0	0.573	0.137
Jurassic	2.7	6.3	16.2	7.8	0.028	0.013
Triassic	2.7	6.3	11.3	8.0	0.019	0.014

Geological period	Glauconite abundance on shelf (wt.%)	Average Si burial rate ($mmol \cdot cm^{-2} \cdot kyr^{-1}$)	World shelf area ($m^2 \cdot 10^{12}$) ‡	2SD	Shallow ocean Si palaeo-flux ($Tmol \cdot yr^{-1}$) ‡	2SD
Holocene	5.6	12.9				
Plio-Pleistocene	5.5	12.6	62.3	0.1	0.428	0.001
Miocene	4.8	11.2	66.4	4.2	0.359	0.023
Oligocene	4.8	11.2	73.3	1.4	0.396	0.008
Eocene	10.9	25.2	78.1	1.4	2.145	0.040
Paleocene	5.5	12.6	78.8	0.6	0.541	0.004
Late Cretaceous	15.6	35.9	82.2	3.1	4.603	0.174
Early Cretaceous	9.9	22.9	75.9	7.5	1.731	0.172
Jurassic	2.7	6.3	68.9	4.9	0.118	0.008
Triassic	2.7	6.3	56.8	2.3	0.098	0.004

Geological period	Glauconite abundance on shelf (wt.%)	Average Fe burial rate ($mmol \cdot cm^{-2} \cdot kyr^{-1}$)	World shelf area ($m^2 \cdot 10^{12}$) †	2SD	Shallow ocean Fe palaeo-flux ($Tmol \cdot yr^{-1}$) †	2SD
Holocene	5.6	4.9	17.9	9.2	0.049	0.025
Plio-Pleistocene	5.5	4.7	15.5	3.5	0.040	0.009
Miocene	4.8	4.2	12.5	1.4	0.025	0.003
Oligocene	4.8	4.2	11.9	1.9	0.024	0.004
Eocene	10.9	9.5	21.4	9.3	0.221	0.096
Paleocene	5.5	4.7	23.6	2.6	0.061	0.007
Late Cretaceous	15.6	13.6	25.5	3.9	0.539	0.081
Early Cretaceous	9.9	8.7	25.1	6.0	0.216	0.052
Jurassic	2.7	2.4	16.2	7.8	0.010	0.005
Triassic	2.7	2.4	11.3	8.0	0.007	0.005

Geological period	Glauconite abundance on shelf (wt.%)	Average Fe burial rate ($mmol \cdot cm^{-2} \cdot kyr^{-1}$)	World shelf area ($m^2 \cdot 10^{12}$) ‡	2SD	Shallow ocean Fe palaeo-flux ($Tmol \cdot yr^{-1}$) ‡	2SD
Holocene	5.6	4.9				
Plio-Pleistocene	5.5	4.7	62.3	0.1	0.161	0.0003
Miocene	4.8	4.2	66.4	4.2	0.135	0.009
Oligocene	4.8	4.2	73.3	1.4	0.149	0.003
Eocene	10.9	9.5	78.1	1.4	0.809	0.015
Paleocene	5.5	4.7	78.8	0.6	0.204	0.002
Late Cretaceous	15.6	13.6	82.2	3.1	1.736	0.065
Early Cretaceous	9.9	8.7	75.9	7.5	0.653	0.065
Jurassic	2.7	2.4	68.9	4.9	0.045	0.003
Triassic	2.7	2.4	56.8	2.3	0.037	0.001

Supplementary References

1. Baldermann, A. et al. The role of Fe on the formation and diagenesis of interstratified glauconite-smectite and illite-smectite: A case study of Upper Cretaceous shallow-water carbonates. *Chem. Geol.* **453**, 21-34 http://dx.doi.org/10.1016/j.chemgeo.2017.02.008 (2017).
2. Wilmsen, M., Niebuhr, B. & Hiss, M. The Cenomanian of northern Germany: facies analysis of a transgressive biosedimentary system. *Facies* **51**, 242-263 https://doi.org/10.1007/s10347-005-0058-5 (2005).
3. Gradstein, F.M., Ogg, J.G. & Smith, A.G. A geologic time scale. Cambridge (University Press) 1-589 (2004).
4. Wilmsen, M. Accommodation- versus capacity-controlled deposition in the Cenomanian (Upper Cretaceous) of northern Germany. *Beringeria* **37**, 239-251 (2007).
5. Wilmsen M. Sequence stratigraphy and palaeoceanography of the Cenomanian Stage in northern Germany. *Cret. Res.* **24**, 525-568 https://doi.org/10.1016/S0195-6671(03)00069-7 (2003).
6. Voigt, T., Wiese, F., von Eynatten, H., Franzke, H.-J. & Gaupp, R. Facies evolution of syntectonic Upper Cretaceous deposits in the Subhercynian Cretaceous Basin and adjoining areas (Germany). *Z. dt. Ges. Geowiss.* **157**, 203-244 https://doi.org/10.1127/1860-1804/2006/0157-0203 (2006).
7. Banerjee, S., Bansal, U. & Thorat, A.V. A review on palaeogeographic implications and temporal variation in glaucony composition. *J. Palaeogeogr.* **5**, 43-71 http://dx.doi.org/10.1016/j.jop.2015.12.001 (2016).
8. Scotese, C.R. & Wright, N. PALEOMAP Paleodigital Elevation Models (PaleoDEMS) for the Phanerozoic. Retrieved from https://www.earthbyte.org/paleodem-resource-scotese-and-wright-2018/ (2018).

9. Wessel, P. et al. The Generic Mapping Tools version 6. Geochem Geophys. 20, 5556-5564 https://doi.org/10.1029/2019GC008515 (2019).

10. Cao, W. et al. Improving global paleogeography since the late Paleozoic using paleobiology. *Biogeosciences* **14**, 5425–5439 https://doi.org/10.5194/bg-14-5425-2017 (2017).

11. Golonka, J., Krobicki, M., Pajak, J., Giang, N. V. & Zuchiewicz, W. Global Plate Tectonics and Paleogeography of Southeast Asia. Faculty of Geology, Geophysics and Environmental Protection, AGH University of Science and Technology, Arkadia, Krakow, Poland (2006).

12. Matthews, K.J. et al. Global plate boundary evolution and kinematics since the late Paleozoic. Global Planet. Change 146, 226-250 https://doi.org/10.1016/j.gloplacha.2016.10.002 (2016).
